# Supplementary figures and images for: Peptidomics insights: neutrophil extracellular traps (NETs) related to the chronic subdural hemorrhage
Source: PeerJ. 2023 Dec 21;11:e16676. doi: 10.7717/peerj.16676 (PMC10749094; doi:10.7717/peerj.16676)

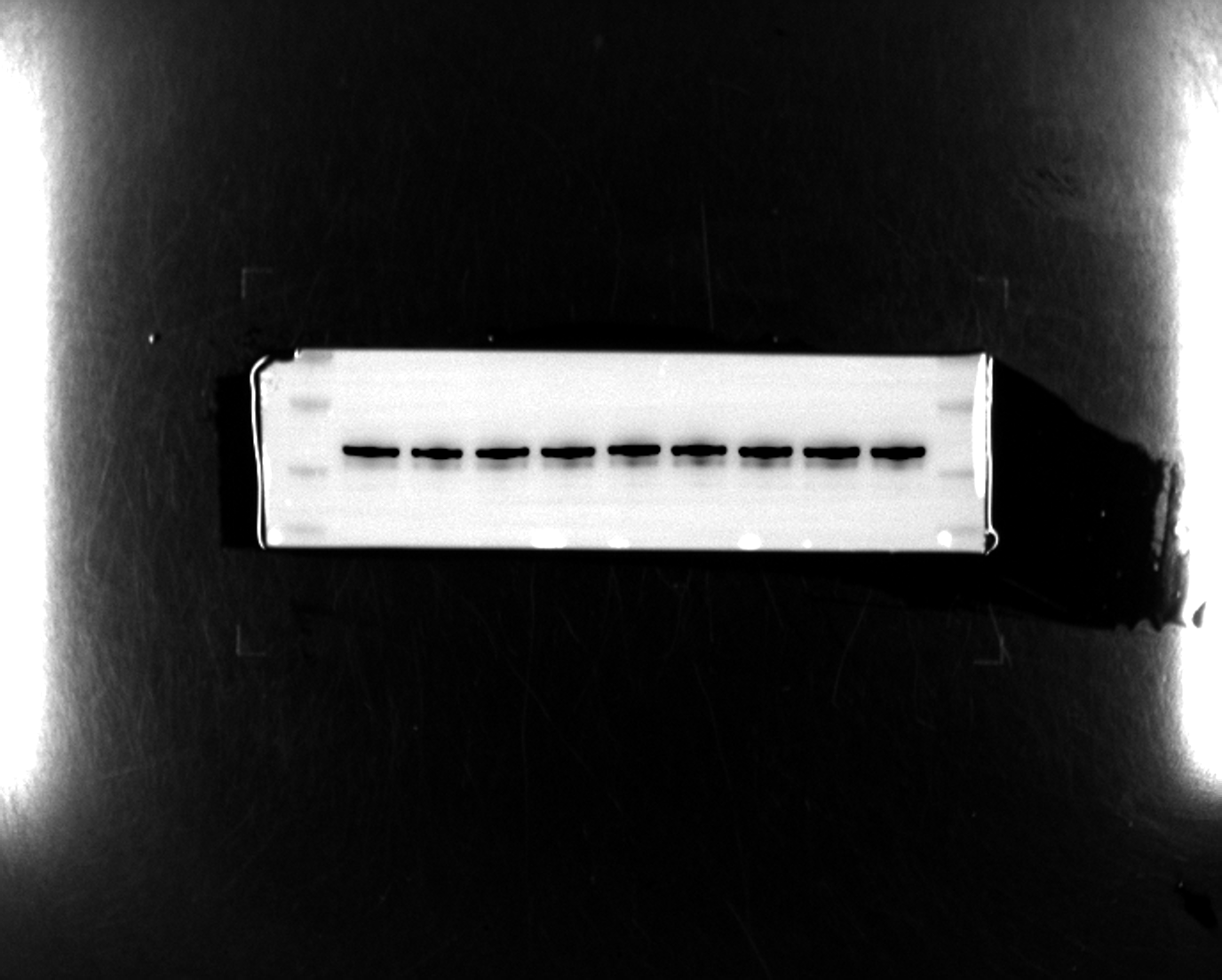

Supplement: Supplemental Information 2 [file peerj-11-16676-s002.zip › Supplementary file2/Actin -2 M.Tif]

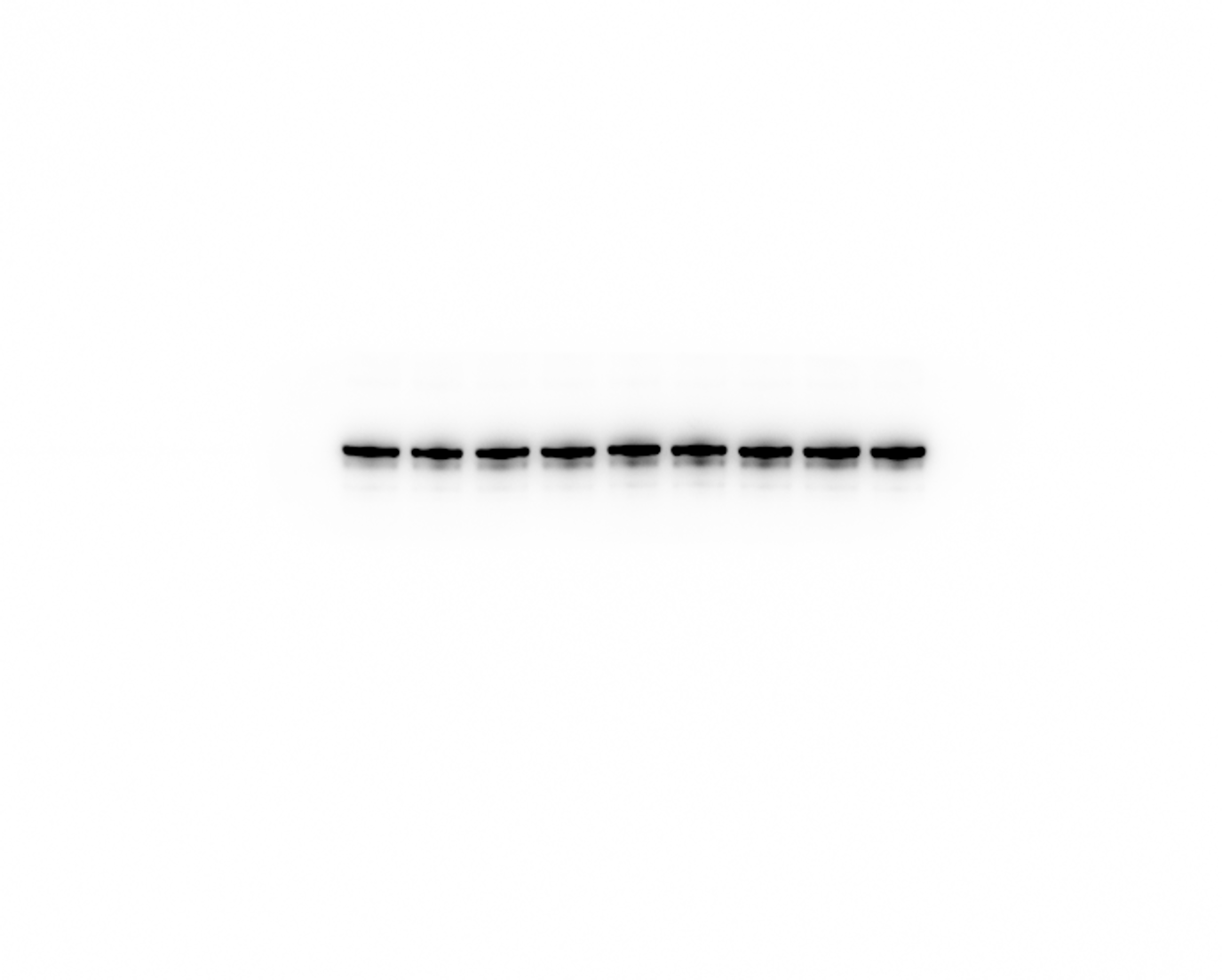

Supplement: Supplemental Information 2 [file peerj-11-16676-s002.zip › Supplementary file2/Actin -2.Tif]

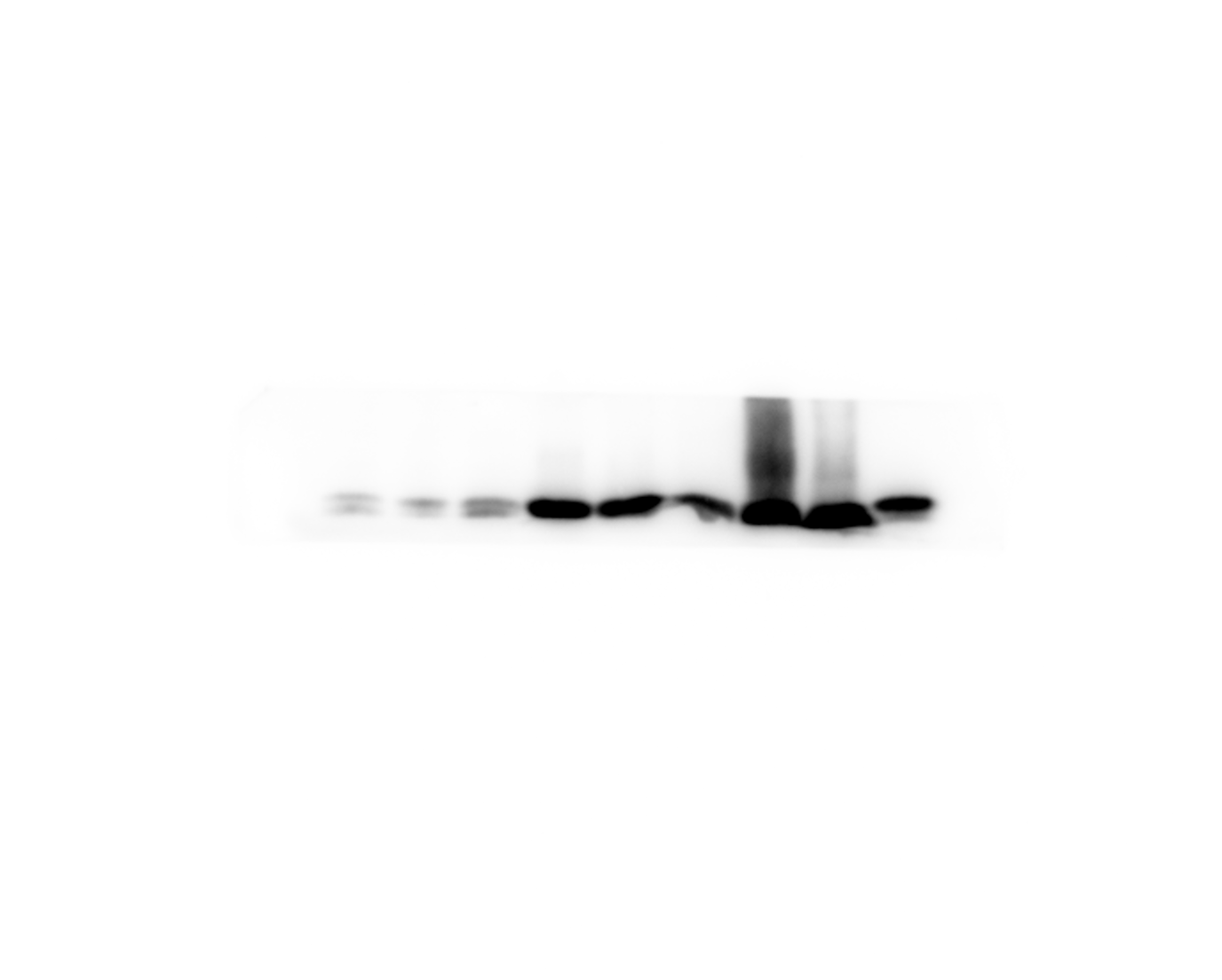

Supplement: Supplemental Information 2 [file peerj-11-16676-s002.zip › Supplementary file2/H3Cit -5.Tif]

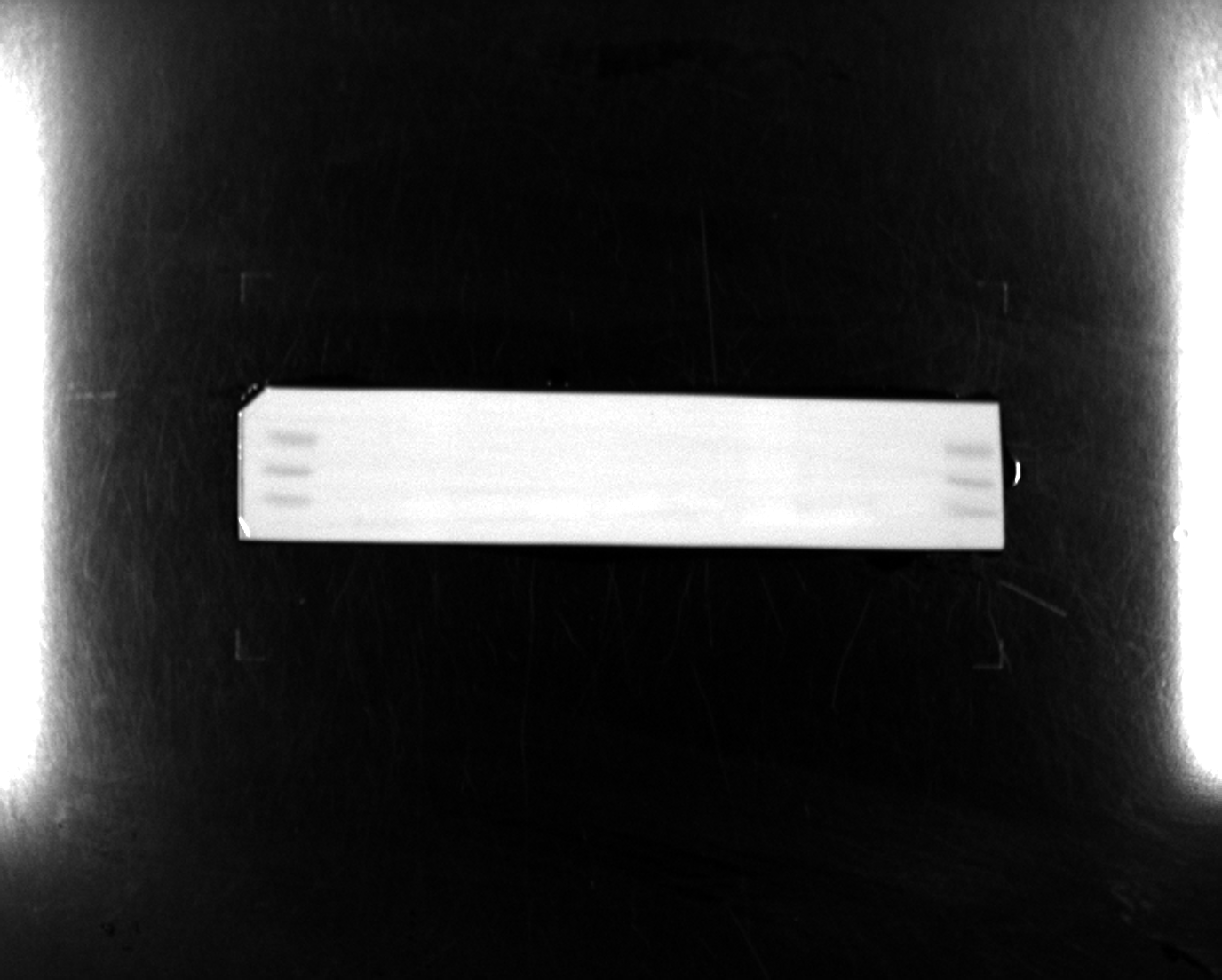

Supplement: Supplemental Information 2 [file peerj-11-16676-s002.zip › Supplementary file2/H3Cit BF.Tif]
